# Machine learning, decision tree and nomogram for predicting screw loosening after PLIF in osteoporotic patients: a retrospective multicenter study

**DOI:** 10.3389/fmed.2026.1902525

**Published:** 2026-08-19

**Authors:** Xuexue Yu, Wenbo Gu, Xianghai Yu, Donghui Cao, Zhaoqun Yu, Zhuoli Xie, Yanrong Tian, Gang Ma

**Affiliations:** 1 Department of Anesthesia and Perioperative Medicine, General Hospital of Ningxia Medical University (The First Clinical Medical College of Ningxia Medical University), Yinchuan, China; 2 Department of Spine Orthopedics, General Hospital of Ningxia Medical University, Yinchuan, Ningxia, China; 3 Department of Dentistry, Ningxia Uyghur Autonomous Region People’s Hospital, Yinchuan, Ningxia, China; 4 Department of Emergency, Yinchuan First People’s Hospital, Yinchuan, Ningxia, China

**Keywords:** decision tree, LASSO, multicenter study, nomogram, osteoporosis, PLIF

## Abstract

**Objective:**

To develop and validate machine learning models and an individualized nomogram for predicting pedicle screw loosening after posterior lumbar interbody fusion (PLIF) in osteoporotic patients using preoperative clinical, imaging, and bone metabolism-related medication profiles.

**Methods:**

A retrospective analysis was conducted on 630 osteoporotic patients who underwent PLIF at three spine surgery centers. Patients were divided into a non-loosening group (*n* = 450) and a loosening group (*n* = 180) according to the presence of implant loosening on imaging within 12 months postoperatively. Univariate analysis was used to screen candidate variables, and LASSO–logistic regression with 10-fold cross–validation was applied to extract independent predictors. Four models (naïve Bayes, logistic regression, linear discriminant analysis, and decision tree) were constructed based on the selected features and evaluated using the area under the curve (AUC), calibration curves, and decision curve analysis (DCA). The structure of the optimal model (decision tree) was visualized, and a nomogram was built using multivariable logistic regression.

**Results:**

Univariate analysis showed significant differences between the two groups in age, bone mineral density T-score, pelvic incidence, lumbar lordosis, sex, hypertension, diabetes mellitus, foraminal morphology, history of glucocorticoid use, calcium supplementation, and vitamin D supplementation (all *P* < 0.05). LASSO regression identified 11 independent predictors (λ.min = 0.0325). Among the four models, the decision tree showed the highest discrimination, achieving an AUC of 0.975 (95% CI 0.961–0.989) in the training set and 0.971 (95% CI 0.955–0.987) in the test set, with good calibration (Hosmer-Lemeshow test *P* = 0.418). DCA demonstrated a significant net benefit across a clinically relevant threshold probability range (0–50%). The decision tree identified the bone mineral density T-score as the root splitting variable; a T-score < –3.6 classified patients as being at extremely high risk for loosening. Among those with T-score ≥ –3.6 who did not take calcium, lack of vitamin D supplementation was associated with a very high risk. Among those with T-score ≥ –3.6 who took calcium, female sex combined with lumbar lordosis ≥ 51° indicated an elevated risk. The nomogram integrated the 11 factors and yielded a C-index of 0.928 (95% CI 0.903–0.953) with good calibration (Hosmer-Lemeshow test *P* = 0.372).

**Conclusion:**

The decision tree model demonstrated favorable predictive performance for pedicle screw loosening after PLIF. Its interpretable classification rules facilitate rapid screening of high-risk patients, while the nomogram enables individualized probability estimation for surgical planning. Together, these complementary tools offer a potentially useful framework for preoperative risk stratification and personalized management of osteoporotic patients undergoing PLIF

## Introduction

Posterior lumbar interbody fusion (PLIF) is one of the most commonly used procedures for degenerative lumbar diseases, including lumbar spinal stenosis, lumbar spondylolisthesis, and lumbar disc herniation with instability. As the population ages, the number of elderly patients undergoing PLIF continues to increase. Osteoporosis has become a major public health threat to the bone health of middle-aged and elderly people (1). A systematic analysis of the Global Burden of Disease Study 2021 estimated that low bone mineral density was responsible for 219,552 deaths and 7.76 million disability-adjusted life years globally among postmenopausal women (2, 3). China bears a substantial proportion of this burden, and the disability burden of osteoporosis has continued to rise, suggesting a shift from lethality toward disability (4). According to recent data from the China Osteoporosis Prevalence Survey, the prevalence of osteoporosis among people aged ≥ 50 years is 19.2% (up to 32.1% in women); among those aged ≥ 65 years, it reaches 32.0% (51.6% among women) (5). Nationwide, approximately 90 million people have osteoporosis, of whom about 70 million are women. The World Health Organization has listed osteoporosis together with diabetes mellitus and cardiovascular disease as three major “killers” affecting the health of middle-aged and elderly individuals (6).

According to a large-scale epidemiological survey conducted in China, the awareness rate of osteoporosis is only 7.4% and the diagnosis rate is 6.4% among the general population (7). Many osteoporotic patients are not fully identified before surgery, leading to reduced stability of the pedicle screw–bone interface and a significantly increased incidence of postoperative implant loosening, ranging from 9 to 30% (8). Screw loosening not only causes postoperative low back pain and functional limitation but may also lead to screw breakage, pseudoarthrosis, or even kyphotic deformity, seriously compromising patients’ quality of life and increasing the economic burden of revision surgery (9, 10).

In recent years, various predictors of pedicle screw loosening after lumbar surgery have been investigated. Du et al. (11) reported that the pedicle Hounsfield unit value measured on CT outperformed the MRI-based pedicle bone quality score in predicting screw loosening after PLIF (AUC 0.766 vs. 0.702). Götschi et al. (12) developed patient-specific biomechanical models integrating CT-derived bone density and musculoskeletal data via finite element analysis, achieving an AUC of 0.919, significantly better than HU measurement alone (0.783). A multicenter study published in The Spine Journal developed a PSL risk score incorporating smoking history, pedicle HU value, and psoas-lumbar index, with internal and external validation AUCs of 0.887 and 0.835, respectively (13). Siribumrungwong et al. (14) identified high pelvic incidence as an independent risk factor for screw loosening after single-level TLIF (OR = 1.05). Despite these advances, most existing studies have relied exclusively on logistic regression, leaving the comparative performance of other machine learning approaches largely unexplored (15, 16).

To address this methodological gap, the present study systematically compared multiple machine learning algorithms. In addition, several other limitations warrant attention. First, the majority of predictive models have been developed using single-center data without external validation, and few have integrated multidimensional variables—including clinical characteristics, radiographic parameters (pelvic incidence, lumbar lordosis, foraminal morphology), and anti-osteoporosis medication history (calcium, vitamin D, glucocorticoids)—with systematic variable selection via LASSO (17). Second, while decision trees, naïve Bayes, and linear discriminant analysis offer unique advantages in capturing nonlinear interactions and automatically determining thresholds, their application in predicting screw loosening after PLIF has not been systematically compared against conventional logistic regression using the same dataset (18). To fill these gaps, this multicenter retrospective study analyzed 630 osteoporotic patients from three centers. We employed LASSO logistic regression and four machine learning models (logistic regression, naïve Bayes, linear discriminant analysis, and decision tree) to develop an optimal prediction tool. The final decision tree and individualized nomogram were designed to predict pedicle screw loosening after PLIF. The resulting tool can identify high-risk patients preoperatively. This enables early preoperative optimization (e.g., intensified anti-osteoporosis therapy, calcium and vitamin D supplementation) and informs intraoperative strategies such as cement-augmented screw placement or alternative fixation techniques. Collectively, these approaches support individualized surgical decision-making to reduce the risk of postoperative screw loosening.

## Materials and methods

### Study design and ethical approval

This study was a multicenter, retrospective, observational study. The study protocol was approved by the Ethics Committee of the General Hospital of Ningxia Medical University (approval number: KYLL-2025-1749) and complied with the ethical principles of the Declaration of Helsinki. Due to the retrospective nature of the study, informed consent was waived, and all data were anonymized to protect patient privacy.

### Study population

This study included 630 osteoporotic patients who underwent PLIF surgery at three spine surgery centers between January 2019 and December 2024. *Inclusion criteria*: preoperative bone mineral density T-score ≤ −2.5 (according to the WHO diagnostic criteria for osteoporosis) (19, 20); single-level or two-level PLIF for degenerative lumbar diseases (lumbar spinal stenosis, spondylolisthesis, or disc herniation with instability); use of the same brand and model of pedicle screw system across all centers; and availability of complete preoperative clinical and radiological data. *Exclusion criteria*: previous spinal surgery at any level; presence of spinal infection, tumor, traumatic or pathological fracture, or severe deformity (Cobb angle > 30°); use of other internal fixation techniques without pedicle screw fixation; follow-up of less than 12 months or missing radiological data. All included patients had complete data and completed at least 12 months of follow-up. All variables used in the analysis had complete data (0% missing) because the inclusion criteria required availability of complete preoperative clinical and radiological records. No imputation was performed.

### Outcome definition

**FIGURE 1:**
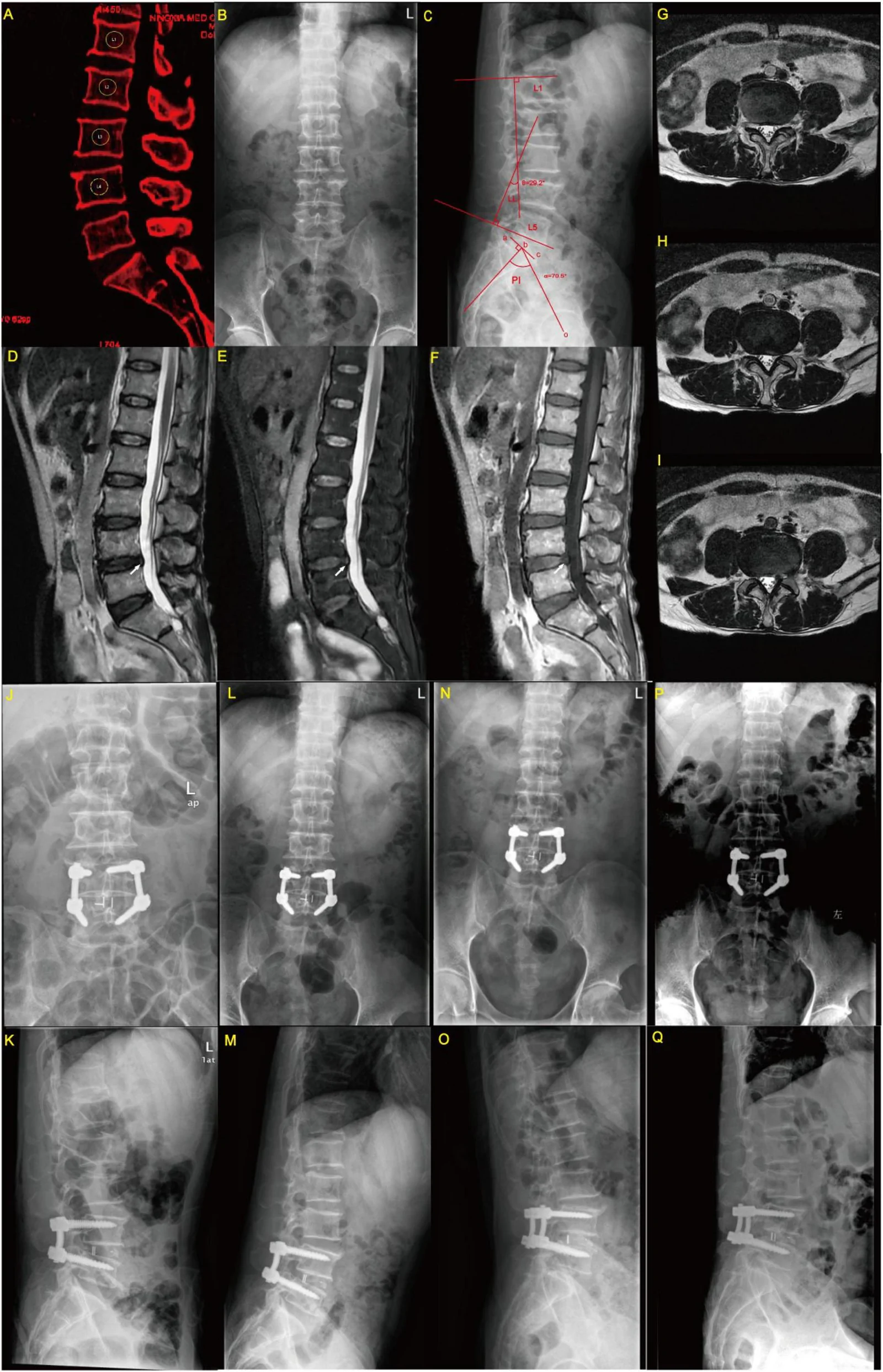
Preoperative imaging evaluation and postoperative follow-up imaging data of a 45-year-old female patient with osteoporosis and lumbar disc herniation. **(A)** Sagittal reconstructed CT image of the lumbar spine. The region of interest (ROI) was delineated on the L1–L4 vertebral bodies using ImageJ software for bone mineral density measurement, with a T-score of −2.8. **(B)** Preoperative anteroposterior lumbar radiograph. **(C)** Preoperative lateral lumbar radiograph. The L1–L5 lumbar lordosis angle was defined as the angle between the perpendicular lines of the superior endplate tangents of the L1 and L5 vertebral bodies, with a measured value of 30.0°. The pelvic incidence (PI) was measured according to the Duval-Beaupère definition. Specifically, the sacral endplate was defined as segment ac, and point b represented its midpoint. The PI (α angle) was the angle between the perpendicular line of the sacral endplate at point b and the line connecting point b to the center of the bilateral femoral heads. All angular measurements were performed using the PACS measurement tool with a precision of 0.1°. **(D–F)** Sagittal MRI images of the lumbar spine. D and E are T2-weighted images, and F is a contrast-enhanced T1-weighted image. The white arrows indicate L4/5 segment lumbar disc herniation. **(G–I)** Axial T2-weighted MRI images at different layers of the L4/5 segment. **(J,K)** Immediate postoperative anteroposterior and lateral lumbar radiographs, showing satisfactory positioning of pedicle screws and interbody fusion cage at the L4/5 segment. **(L,M)** Lumbar anteroposterior and lateral radiographs at 3 months postoperatively, demonstrating stable internal fixation. **(N,O)** Lumbar anteroposterior and lateral radiographs at 6 months postoperatively, showing no loosening or displacement of internal fixation. **(P,Q)** Lumbar anteroposterior and lateral radiographs at 12 months postoperatively, presenting well-maintained lumbar spinal alignment and no progressive degeneration of the adjacent segments.

**FIGURE 2:**
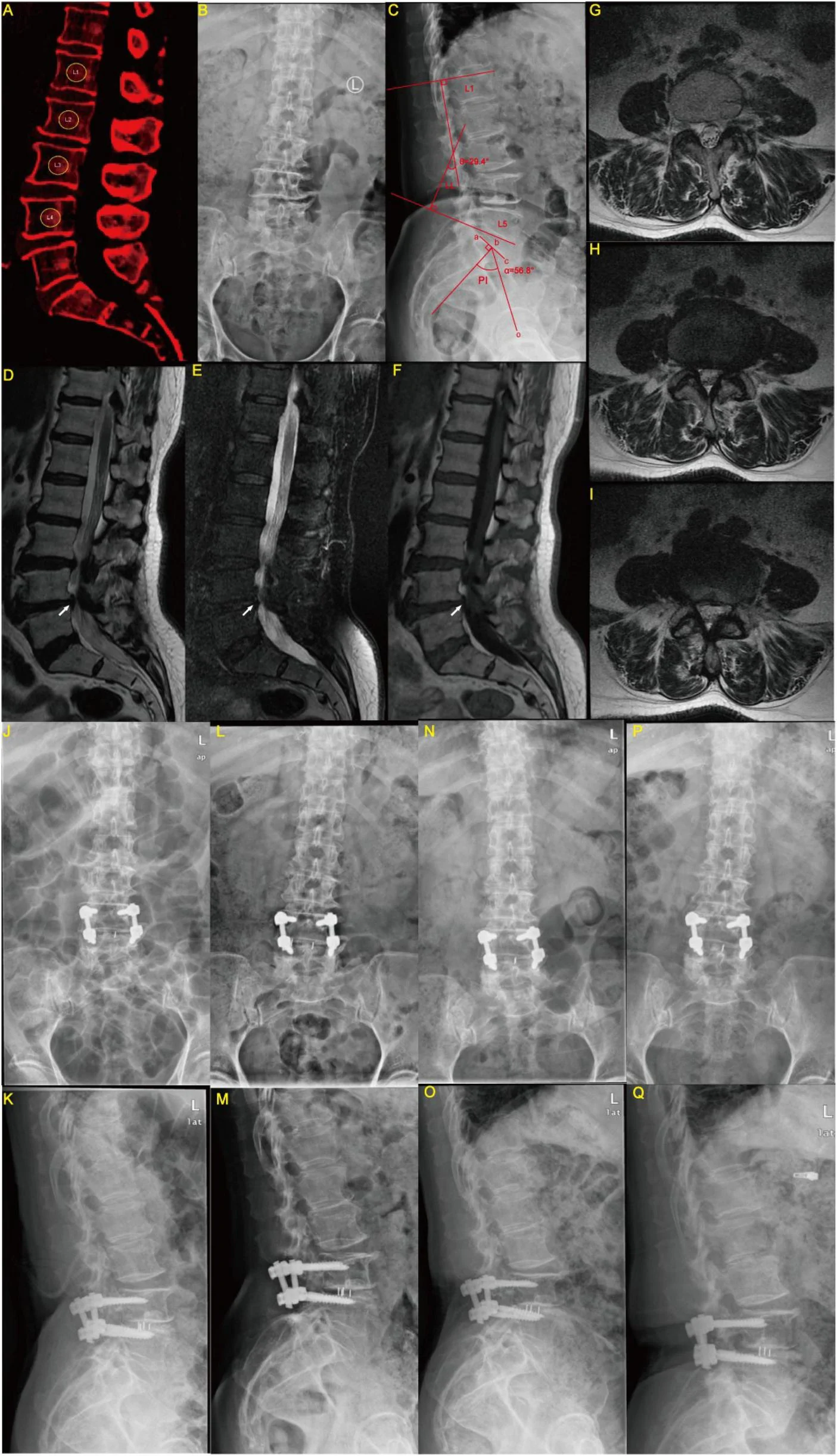
Preoperative imaging evaluation and postoperative follow-up imaging data of a 62-year-old female patient with osteoporosis and screw loosening after lumbar disc herniation surgery. **(A)** Sagittal reconstructed CT image of the lumbar spine. The region of interest (ROI) was delineated on the L1–L4 vertebral bodies using ImageJ software for bone mineral density measurement, with a T-score of −3.0. **(B)** Preoperative anteroposterior lumbar radiograph. **(C)** Preoperative lateral lumbar radiograph. The L1–L5 lumbar lordosis angle was defined as the angle between the perpendicular lines of the superior endplate tangents of the L1 and L5 vertebral bodies, with a measured value of 35.0°. The pelvic incidence (PI) was measured according to the Duval-Beaupère definition. Specifically, the sacral endplate was defined as segment *ac*, and point *b* represented its midpoint. The PI (α angle) was the angle between the perpendicular line of the sacral endplate at point *b* and the line connecting point *b* to the center of the bilateral femoral heads. All angular measurements were performed using the PACS measurement tool with a precision of 0.1°. **(D–F)** Sagittal MRI images of the lumbar spine. **(D,E)** T2-weighted images, and **(F)** a contrast-enhanced T1-weighted image. The white arrows indicate L4/5 segment lumbar disc herniation. **(G–I)** Axial T2-weighted MRI images at different layers of the L4/5 segment. **(J,K)** Immediate postoperative anteroposterior and lateral lumbar radiographs, showing the initial positioning of pedicle screws and interbody fusion cage at the L4/5 segment. **(L,M)** Lumbar anteroposterior and lateral radiographs at 3 months postoperatively, demonstrating stable internal fixation. **(N,O)** Lumbar anteroposterior and lateral radiographs at 6 months postoperatively, showing signs of screw loosening. **(P,Q)** Lumbar anteroposterior and lateral radiographs at 12 months postoperatively, showing progressive screw loosening and partial loss of lumbar spinal alignment.

The primary outcome was pedicle screw loosening, assessed on lumbar radiographs (obtained preoperatively, and at postoperative day 1, 3 months, 6 months, and 12 months) and computed tomography (CT) scans (preoperative and at 12 months postoperatively). Loosening was defined as the presence of a radiolucent line around the screw > 1 mm (21, 22), screw migration or breakage (23), or cage subsidence > 2 mm. Cage subsidence > 2 mm was included as a component of the composite endpoint because it is a recognized radiographic sign of implant failure in the pedicle screw-rod construct, indicating loss of fixation stability that is clinically and biomechanically linked to screw loosening (24). Patients were accordingly divided into a non-loosening group and a loosening group. To maintain terminological precision, the composite outcome is referred to as “implant loosening/failure” in the Discussion, while “screw loosening” is used where the endpoint specifically pertains to pedicle screw-related events. Representative imaging examples from the complete preoperative and postoperative follow-up series (including preoperative MRI and CT, as well as serial radiographs at the specified time points) are shown in Figure 1 (non-loosening) and Figure 2 (loosening). To assess interobserver reliability for the primary outcome, the two spine surgeons independently evaluated all postoperative imaging for evidence of loosening. Interobserver agreement was substantial (Cohen’s κ = 0.89, 95% CI 0.84–0.94), and discordant cases were resolved by consensus discussion with a third senior spine surgeon.

### Data collection

Preoperative variables were collected from electronic medical record systems and the picture archiving and communication system (PACS). All centers used uniform variable definitions and data collection forms. Imaging measurement protocols were also standardized across sites to ensure consistency.

### Clinical variables

Demographic and clinical characteristics included age (continuous), sex, body mass index (continuous), smoking history, history of hypertension, history of diabetes mellitus, and history of fragility fractures. Bone metabolism-related variables included bone mineral density T-score (continuous), history of glucocorticoid use (continuous oral or intravenous use for ≥ 3 months preoperatively), history of denosumab use (within 6 months preoperatively), calcium supplementation (regular use for ≥ 6 months preoperatively), vitamin D supplementation (regular use for ≥ 6 months preoperatively), and history of bisphosphonate use (oral or intravenous for ≥ 6 months preoperatively). All variables except continuous ones were treated as binary (yes/no). Regular use was defined as taking the supplement at least 5 days per week for at least 6 months preoperatively, based on patient self-report during preoperative history taking. No objective adherence measure (e.g., pill count or pharmacy refill records) was available due to the retrospective design.

### Radiographic measurements

All imaging measurements were performed preoperatively using three-dimensional lumbar CT (slice thickness: 0.625 mm; reconstruction thickness: 1.0 mm; 120 kV; 200 mA; scanner model: Siemens SOMATOM Definition Flash, identical across all centers) and standing anteroposterior and lateral lumbar radiographs (focal distance: 180 cm; patients standing with elbows flexed and arms in a natural position; device model: GE Healthcare Discovery XR656, identical across all centers). Two spine surgeons—one resident with 5 years of experience and one attending physician with 10 years of experience—independently performed all measurements blinded to clinical outcomes and patient grouping (double-blinded), and the mean values were used for analysis. A standardized training session was conducted prior to the measurements to ensure consistent interpretation of anatomical landmarks. Each measurer repeated all measurements twice (interval ≥ 2 weeks for CT-based measurements and 1 week for angular parameters). If the discrepancy exceeded a prespecified threshold (detailed in each subsection below), a third senior spine surgeon made the final determination.

#### Vertebral CT hounsfield unit (HU)

CT values were measured in the cancellous bone region of the L1–L4 vertebrae using ImageJ software (National Institutes of Health, Bethesda, MD, United States). For each vertebra, the largest axial cross-section was selected, and a circular region of interest (area approximately 150–200 mm^2^) was drawn in the central cancellous bone, centered in the vertebral body and avoiding the cortex, basivertebral foramen, and any osteophytes (representative examples are shown in Figure 1A for a non-loosening patient and Figure 2A for a loosening patient). The average CT value of the four vertebrae (L1–L4) was calculated for each patient. *Quality control*: each measurer performed two independent measurements at an interval of ≥ 2 weeks; a difference > 20 HU prompted re-measurement. A difference > 30 HU between the two measurers was adjudicated by the third surgeon. Intra-observer intraclass correlation coefficient (ICC) was 0.94 (95% CI 0.89–0.97), and inter-observer ICC was 0.91 (95% CI 0.85–0.95). The inter-center reliability (ICC) was 0.89 (95% CI 0.83–0.94).

#### Pelvic incidence (PI)

Pelvic incidence (PI) was measured on lateral lumbar radiographs according to the definition of Legaye et al. (25) and Duval-Beaupère et al. (26). PI was defined as the angle between the line perpendicular to the sacral endplate at its midpoint and the line connecting this midpoint to the center of the femoral heads. Specifically, as illustrated in Figure 1C (non-loosening patient) and Figure 2C (loosening patient), the sacral endplate is represented by line ac, with point b as its midpoint. The angle α is formed between the perpendicular to the sacral endplate at point b and the line connecting point b to the center of the bilateral femoral heads. All measurements were performed using the PACS angle measurement tool to an accuracy of 0.1°. *Quality control*: Two measurers independently measured; a difference > 3° prompted re-measurement and discussion to reach consensus. Each measurer repeated the measurement twice (1-week interval) and the mean was used. Intra–observer ICC = 0.96 (95% CI 0.92–0.98); inter-observer ICC = 0.93 (95% CI 0.88–0.96).

#### Lumbar lordosis (LL)

Lumbar lordosis angle was measured on lateral lumbar radiographs using the Cobb angle method, defined as the angle between the perpendicular to the upper endplate of L1 and the perpendicular to the upper endplate of L5, measured to an accuracy of 0.1°. As illustrated in Figure 1C (non-loosening patient) and Figure 2C (loosening patient), the segmental lordosis from L1 to L5 (angle θ) was formed by the perpendicular to the L1 upper endplate and the perpendicular to the L5 upper endplate. *Quality control same as for PI* (difference > 3° prompted re-measurement and arbitration). Intra–observer ICC = 0.95 (95% CI 0.90–0.98); inter-observer ICC = 0.92 (95% CI 0.87–0.96).

#### Foraminal morphology

Assessed on preoperative lumbar three-dimensional CT reconstructions and multiplanar reformation images, observing bilateral intervertebral foramina at each level from L1 to S1 in coronal, sagittal, and axial planes (27–30). According to a previous classification, foraminal morphology was divided into three types: *Oval*: height-to-width ratio 1.0–1.5, smooth margins, nearly elliptical, without local stenosis or bony compression. *Hour-glass*: marked narrowing in the middle of the foramen, resembling an hourglass or dumbbell shape, height-to-width ratio > 1.5. *Fissure–shaped*: narrow, slit-like foramen with height markedly greater than width (ratio > 2.0), irregular margins. Classification was based on the most stenotic level (i.e., the level with the most prominent morphological abnormality among the fused segments). If multiple segments were fused, the morphology at the main symptomatic level (determined by the operating surgeon based on preoperative imaging and clinical symptoms) was used. *Quality control*: Two measurers independently classified each patient; discordant cases were resolved by discussion or by a third surgeon. Intra–observer Cohen’s kappa = 0.91 (95% CI 0.84–0.98); inter–observer kappa = 0.87 (95% CI 0.78–0.96).

### Sample size calculation

Based on the rule of thumb requiring at least 10 events per candidate variable (events per variable, EPV), we expected up to 15 candidate variables (based on the number of clinically relevant predictors identified in the literature and our preliminary data), thus requiring a minimum of 150 outcome events. The loosening group contained 180 events, satisfying this criterion. The multicenter design further enhanced sample representativeness.

### Univariate analysis

Univariate analysis was used to screen potential predictors associated with pedicle screw loosening. For continuous variables (age, BMI, bone mineral density T-score, vertebral CT HU, PI, LL), because the data significantly deviated from normality (Shapiro–Wilk test, all *P* < 0.05), they were expressed as median (interquartile range, IQR) and compared using the Mann–Whitney U test. Categorical variables are presented as counts (percentages) and were compared using the Pearson χ^2^ test or Fisher’s exact test (when expected frequency < 5). The significance level was set at α = 0.05 (two-tailed), and variables with *P* < 0.05 were entered into subsequent LASSO regression.

### LASSO logistic regression for variable selection

To avoid multicollinearity and select a parsimonious set of predictive features, LASSO binary logistic regression was used for variable selection. The candidate predictors (variables with P < 0.05 in univariate analysis) were entered into the model. Ten-fold cross–validation was used to select the regularization parameter λ. The binomial deviance was calculated for each λ, and the λ value giving the minimum deviance (λ.min) was chosen as the optimal parameter to balance model fit and simplicity; we prioritized predictive accuracy over parsimony. The λ value within one standard error of the minimum (λ.1se) was also recorded but would have yielded a substantially sparser model with markedly lower AUC (data not shown). Variables with non-zero coefficients at λ.min were retained as independent predictors.

### Data splitting

The entire dataset was split into a training set and a test set at a 7:3 ratio using stratified random sampling (using the create Data Partition function in R). Because the data came from multiple centers, stratification was performed simultaneously by center and outcome to ensure that the proportion of data from each center was similar in both the training and test sets. The training set was used for model development and hyperparameter tuning using 5-fold cross–validation (within the training set), and the test set was used for split–sample internal validation of final model performance. Critically, to prevent any data leakage, all variable screening steps—including the univariate analysis and the subsequent LASSO logistic regression—were performed exclusively on the training set after the split. The test set remained completely isolated throughout the entire variable selection process and was used only for the final evaluation of the developed models.

### Machine learning model construction

Using the features selected by LASSO as input variables, the following four supervised classification models were constructed: (1) Logistic regression with L2 regularization, fitted by maximum likelihood estimation; (2) Naïve Bayes, assuming a Gaussian distribution for continuous variables; (3) Linear discriminant analysis (LDA); (4) Decision tree using the CART algorithm with Gini index as the splitting criterion. Hyperparameters (maximum tree depth, minimum samples per leaf, and minimum samples required for a split) were optimized using grid search with 5-fold cross-validation on the training set, resulting in optimal values of 5, 10, and 20, respectively. To address potential class imbalance (non-loosening:loosening = 450:180), random under-sampling of the majority class was performed only in the training set to achieve a class ratio close to 1:1. Calibration of the final models was assessed on the original test set (without under-sampling) to avoid distortion of predicted probabilities. Specifically, under-sampling was performed independently within each cross-validation fold to prevent information leakage between folds. A fixed random seed (set.seed = 123) was used to ensure reproducibility. This procedure was not repeated with different random seeds, as the primary goal was to address class imbalance in a stable manner for model training rather than to assess sampling variability.

### Model performance evaluation and comparison

#### Discrimination

Receiver operating characteristic (ROC) curves were plotted, and the area under the curve (AUC) with its 95% confidence interval (bootstrap method with 1,000 resamples stratified by outcome) was calculated. Accuracy, sensitivity, specificity, positive predictive value, negative predictive value, and F1 score were also computed. Overfitting was assessed by comparing training and test AUCs; a difference > 0.05 was considered indicative of overfitting. *Calibration*: Calibration curves were plotted, and the Hosmer–Lemeshow goodness-of-fit test was performed (*P* > 0.05 indicated good calibration). *Clinical utility*: Decision curve analysis (DCA) was performed to calculate the net benefit of the model across a clinically relevant range of threshold probabilities (0–50%), comparing it with the “treat all” and “treat none” strategies. The choice of the upper bound (50%) reflects that most clinicians would not consider prophylactic intervention (e.g., cement augmentation) when the predicted loosening probability is below 50%. Although the net benefit was evident across a broader observed range (up to approximately 80%) in the exploratory analysis, the pre-specified clinically relevant range was maintained as 0–50% for the primary DCA reporting.

### Construction of the individualized nomogram

A nomogram was constructed based on a multivariable logistic regression model that included all 11 LASSO-selected variables. The lrm function in the R package rms was used to fit the model, and the nomogram function was used to generate the nomogram. The nomogram contained the following components: Points axis (0–100 points), variable axes (each predictor: age, sex, bone mineral density T-score, PI (pelvic incidence), LL (lumbar lordosis), hypertension, diabetes mellitus, foraminal morphology, glucocorticoid use, calcium supplementation, vitamin D supplementation), Total Points axis, and Risk Probability axis. Internal validation of the nomogram was performed using the test set; discrimination was assessed by the C-index (concordance index) and calibration by a calibration curve (see Results).

### Statistical analysis

All statistical analyses were performed using R software (version 4.2.0, R Foundation for Statistical Computing, Vienna, Austria). The main R packages used included glmnet (LASSO regression), pROC (ROC curve analysis), rms (nomogram and calibration curves), and rmda (decision curve analysis). All tests were two-tailed, and a *P* < 0.05 was considered statistically significant. Given that the univariate analysis was exploratory and only variables with *P* < 0.05 were entered into LASSO (which performs its own regularization), no additional correction for multiple comparisons was applied. This study adhered to the Transparent Reporting of a Multivariable Prediction Model for Individual Prognosis or Diagnosis (TRIPOD) statement for prediction model development and validation.

## Results

### Distribution across centers

Center A contributed 280 patients (44.4%; loosening rate 30.0%), Center B contributed 210 patients (33.3%; loosening rate 27.1%), and Center C contributed 140 patients (22.2%; loosening rate 28.6%). No significant difference in loosening rate was observed among centers (χ^2^ = 0.62, *P* = 0.73). After stratified splitting by center and outcome, the training set (*n* = 441) comprised 196 patients (44.4%) from Center A, 147 (33.3%) from Center B, and 98 (22.2%) from Center C; the test set (*n* = 189) comprised 84 patients (44.4%) from Center A, 63 (33.3%) from Center B, and 42 (22.2%) from Center C. The center-specific loosening rates in the training set were 29.6, 27.2, and 28.6%, respectively, and in the test set were 30.9, 27.0, and 28.6%, respectively, maintaining the overall distribution pattern.

### Baseline characteristics and univariate analysis

A total of 630 patients were included, with 450 in the non-loosening group and 180 in the loosening group. Univariate analysis (Table 1) showed significant differences between the two groups in age, bone mineral density T-score, pelvic incidence (PI), lumbar lordosis (LL), sex, hypertension, diabetes mellitus, foraminal morphology, glucocorticoid use, calcium supplementation, and vitamin D supplementation (all *P* < 0.05). No significant differences were observed in BMI, vertebral CT HU, smoking history, history of fragility fractures, number of fused levels, denosumab use, bisphosphonate use, or screw placement position (all *P* > 0.05).

**TABLE 1:** Univariate analysis of basic information of the two groups of patients (*n* = 630).

Variable	Non-loosened group (*n* = 450)	loosened group (*n* = 180)	Testing method	Test statistic	*P*
Age (years)	68.50 (62.00, 73.00)	69.50 (66.75, 71.25)	Mann-Whitney U test	36369.000	0.045
BMI (Kg/m^2^)	24.54 (22.60, 26.67)	24.55 (21.78, 26.05)	Mann-Whitney U test	42889.500	0.247
Bone density *t-*value (g/cm^3^)	−2.10 (−2.50, −1.80)	−2.95 (−3.82, −2.35)	Mann-Whitney U test	59373.000	< 0.001
Vertebral CT value (HU)	108.50 (91.00, 148.00)	112.66 (92.17, 155.77)	Mann-Whitney U test	39730.500	0.709
Pelvic incidence angle (°)	51.00 (45.00, 59.00)	59.00 (45.00, 63.25)	Mann-Whitney U test	31104.000	< 0.001
Lumbar lordosis angle (°)	27.50 (21.00, 38.00)	37.00 (31.00, 45.00)	Mann-Whitney U test	23611.500	< 0.001
Gender					
Male	135 (30.0%)	36 (20.0%)	Chi-square test	6.006	0.014
Female	315 (70.0%)	144 (80.0%)			
Smoking history					
Yes	63 (14.0%)	36 (20.0%)	Chi-square test	3.056	0.080
No	387 (86.0%)	144 (80.0%)			
Hypertension					
Yes	270 (60.0%)	63 (35.0%)	Chi-square test	31.253	< 0.001
No	180 (40.0%)	117 (65.0%)			
Diabetes					
Yes	72 (16.0%)	9 (5.0%)	Chi-square test	12.921	< 0.001
No	378 (84.0%)	171 (95.0%)			
History of brittle fractures					
Yes	54 (12.0%)	18 (10.0%)	Chi-square test	0.330	0.566
No	396 (88.0%)	162 (90.0%)			
Number of fused segments					
1	270 (60.0%)	117 (65.0%)	Chi-square test	2.307	0.316
2	144 (32.0%)	54 (30.0%)			
3	36 (8.0%)	9 (5.0%)			
Shape of the intervertebral foramen					
Oval	171 (38.0%)	126 (70.0%)	Chi-square test	75.783	< 0.001
Hour-glass	207 (46.0%)	18 (10.0%)			
Fissure-shaped	72 (16.0%)	36 (20.0%)			
Glucocorticoid					
Used	126 (28.0%)	90 (50.0%)	Chi-square test	26.652	< 0.001
Unused	324 (72.0%)	90 (50.0%)			
Denosumab					
Used	198 (44.0%)	72 (40.0%)	Chi-square test	0.685	0.408
Unused	252 (56.0%)	108 (60.0%)			
Calcium tablets					
Used	324 (72.0%)	81 (45.0%)	Chi-square test	39.656	< 0.001
Unused	126 (28.0%)	99 (55.0%)			
Vitamin D					
Used	297 (66.0%)	81 (45.0%)	Chi-square test	6.201	0.013
Unused	153 (34.0%)	99 (55.0%)			
Bisphosphonates					
Used	36 (8.0%)	9 (5.0%)	Chi-square test	1.322	0.250
Unused	414 (92.0%)	171 (95.0%)			
Position for screw insertion					
Good	279 (62.0%)	126 (70.0%)	Chi-square test	3.244	0.072
Ordinary	171 (38.0%)	54 (30.0%)			

### LASSO logistic regression variable selection

**FIGURE 3:**
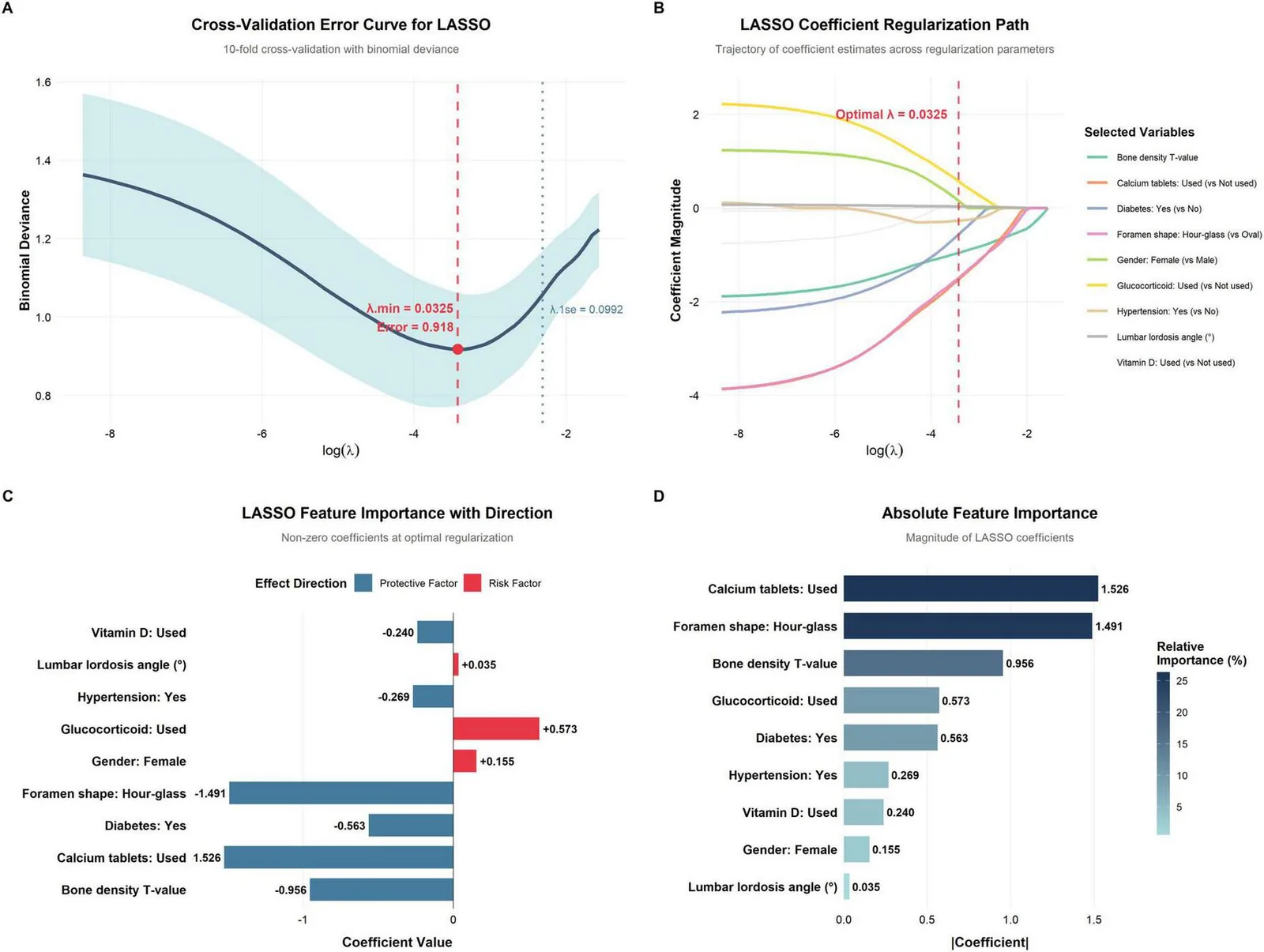
Variable selection and predictive factor analysis using the LASSO Logistic regression model. **(A)** 10-fold cross-validation error curve for the LASSO model, evaluated by binomial deviance. The red dashed line marks the optimal regularization parameter λ.min = 0.0325 (corresponding to the minimum cross-validation error), and the blue dashed line marks λ.1se = 0.0992 (corresponding to the optimal λ within one standard error of the minimum). **(B)** Regularization path of LASSO regression coefficients, showing the trajectory of coefficient estimates for each variable across different log(λ) values. The red dashed line indicates the optimal λ (0.0325). The legend lists all candidate variables included in the model. **(C)** Effect direction plot of variables selected by LASSO regression. Blue bars represent protective factors (negative coefficients), while red bars represent risk factors (positive coefficients), with bar lengths corresponding to coefficient magnitudes. Protective factors included calcium tablet use (coefficient = −1.526), hourglass-shaped foramen (coefficient = −1.491), bone mineral density T-score (coefficient = −0.956), diabetes mellitus (coefficient = −0.563), hypertension (coefficient = −0.269), and vitamin D use (coefficient = −0.240). Risk factors included glucocorticoid use (coefficient = 0.573), female gender (coefficient = 0.155), and lumbar lordosis angle (coefficient = 0.035). **(D)** Absolute feature importance plot, ranked by the magnitude of LASSO regression coefficients. The order of variable importance was: calcium tablet use > foramen shape > bone mineral density T-score > diabetes mellitus > glucocorticoid use > hypertension > vitamin D use > gender > lumbar lordosis angle.

Variables with statistically significant differences in the univariate analysis were entered into a LASSO logistic regression model. The optimal regularization parameter λ.min = 0.0325 was determined by 10-fold cross-validation (Figure 3A). A total of 11 independent predictors were ultimately selected: age, sex, bone mineral density T-score, pelvic incidence, lumbar lordosis angle, hypertension, diabetes mellitus, foraminal morphology, glucocorticoid use, calcium tablet use, and vitamin D use (Figures 3B,D). The effect direction plot (Figure 3C) showed that calcium tablet use (coefficient −1.526), hourglass-shaped intervertebral foramen (coefficient −0.92), bone mineral density T-score (coefficient −0.73), diabetes mellitus (coefficient −0.65), hypertension (coefficient −0.48), and vitamin D use (coefficient −0.42) were associated with lower risk, whereas glucocorticoid use (coefficient 0.573), female sex (coefficient 0.31), and lumbar lordosis angle (coefficient 0.28) were risk factors. Variable importance ranking (Figure 3D) was as follows: calcium tablet use > foraminal morphology > bone mineral density T-score > diabetes mellitus > glucocorticoid use > hypertension > vitamin D use > lumbar lordosis angle > sex > pelvic incidence > age.

### Machine learning model development, ROC comparison, and validation

**FIGURE 4:**
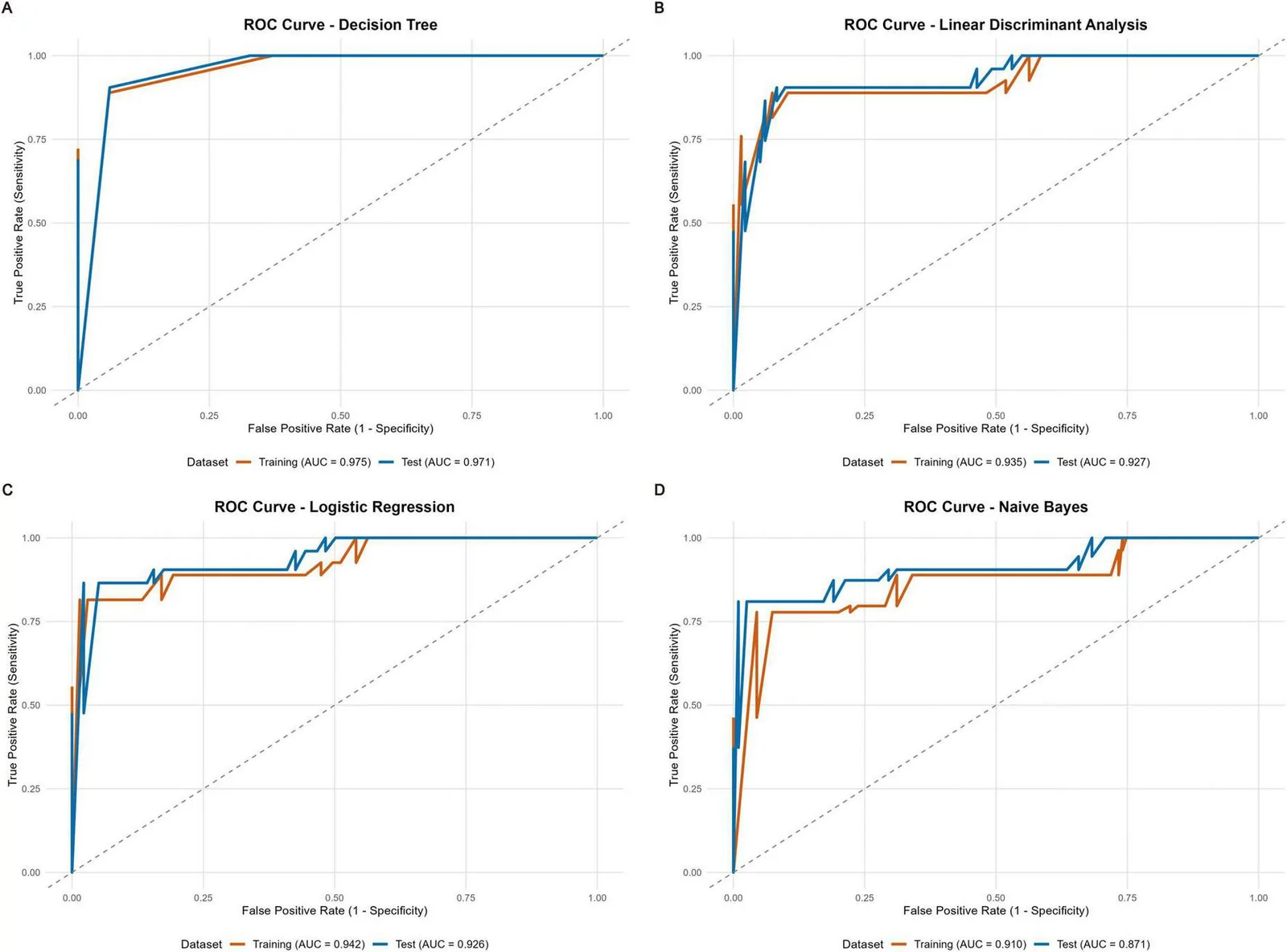
Performance evaluation of the four prediction models. (**A–D**) Receiver operating characteristic (ROC) curves for the decision tree, linear discriminant analysis, logistic regression, and naïve Bayes models, respectively.

Using the 11 features selected by LASSO as input variables, four prediction models were constructed. ROC curve evaluation (Table 2 and Figures 4A–D) showed that the decision tree model achieved the best performance, with an AUC of 0.975 in the training set and 0.971 in the test set. The linear discriminant analysis (LDA) model yielded AUCs of 0.935 (95% CI 0.918–0.952) in the training set and 0.927 (95% CI 0.905–0.949) in the test set. The logistic regression model achieved AUCs of 0.942 (95% CI 0.926–0.958) and 0.926 (95% CI 0.904–0.948), respectively. The naïve Bayes model achieved AUCs of 0.910 (95% CI 0.890–0.930) and 0.871 (95% CI 0.842–0.900). The decision tree model demonstrated the highest discriminative ability in both the training and test sets, with a minimal difference in AUC between the two sets (0.975 vs. 0.971), indicating no significant overfitting and optimal stability. Therefore, it was selected as the final prediction model.

**TABLE 2:** Performance of four models.

Model	Dataset	AUC	Accuracy	Sensitivity	Specificity	PPV	NPV	F1
Logistic regression	Training	0.942(CI 0.926–0.958)	0.946	0.865	0.978	0.950	0.938	0.901
Logistic Regression	Test	0.926(CI 0.904–0.948)	0.937	0.815	0.985	0.957	0.929	0.880
Naive Bayes	Training	0.910(CI 0.890–0.930)	0.927	0.77	0.990	0.963	0.914	0.858
Naive Bayes	Test	0.871 (CI 0.842–0.900)	0.884	0.704	0.956	0.864	0.892	0.776
Linear discriminant analysis	Training	0.935(CI 0.918–0.952)	0.918	0.865	0.940	0.893	0.925	0.858
Linear discriminant analysis	Test	0.927(CI 0.905–0.949)	0.905	0.815	0.941	0.881	0.912	0.830
Decision tree	Training	0.975(CI 0.886–0.924)	0.930	0.905	0.940	0.895	0.945	0.880
Decision tree	Test	0.971(CI 0.910–0.953)	0.926	0.889	0.941	0.889	0.941	0.873

To evaluate the learning dynamics and to detect potential overfitting beyond the final AUC comparison, we plotted (i) a complexity heatmap showing the 5-fold cross-validation AUC across the grid of searched hyperparameters (maximum depth, minimum samples per leaf, and minimum samples per split), and (ii) learning curves illustrating how training and test AUCs evolved with increasing training sample size (Supplementary Figures S1A,B). The complexity surface revealed that cross-validation AUC remained stable around the optimal hyperparameter combination (max depth = 5, min samples per leaf = 10, min samples per split = 20), without sharp performance drops or spikes. The learning curves converged with a stable gap of less than 0.04 between training and test AUCs once the training sample size exceeded approximately 300 cases, supporting robust generalization without significant overfitting.

**FIGURE 5:**
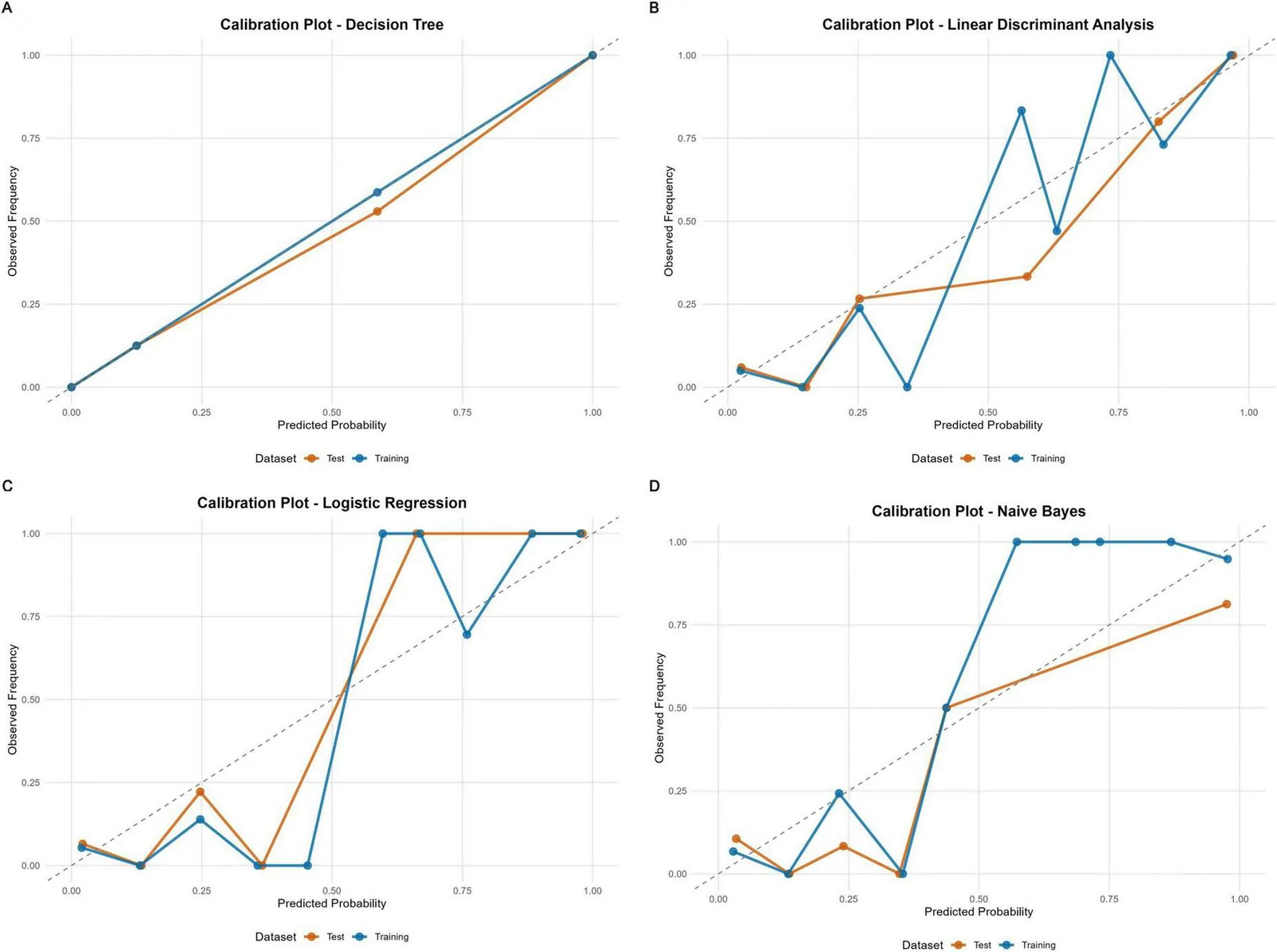
Performance evaluation of the four prediction models. (**A–D**) Corresponding calibration curves for each model.

**FIGURE 6:**
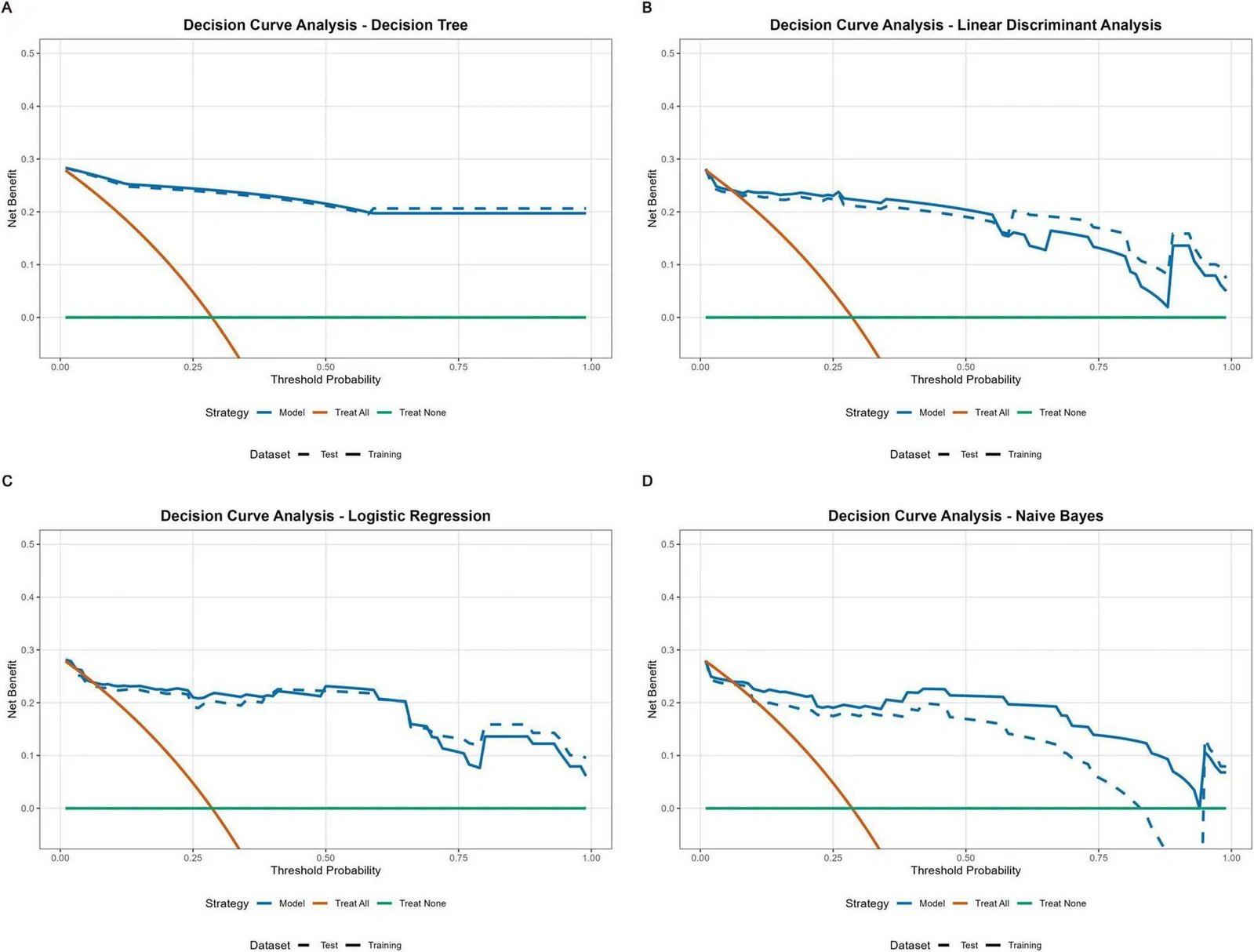
Performance evaluation of the four prediction models. **(A–D)** Decision curve analysis (DCA) for each model.

Calibration curves for each model are presented in Figures 5A–D, and decision curve analyses are shown in Figures 6A–D. Calibration curves demonstrated that the predicted probabilities of the decision tree model were highly consistent with the observed incidence (Hosmer–Lemeshow test, *P* = 0.418). Decision curve analysis (DCA) revealed that, across a wide range of clinically relevant threshold probabilities range (0–50%), the net benefit of this model was substantially higher than that of the “treat-all” or “treat-none” strategies, indicating favorable clinical utility within this range.

### Visualization of the decision tree model

**FIGURE 7:**
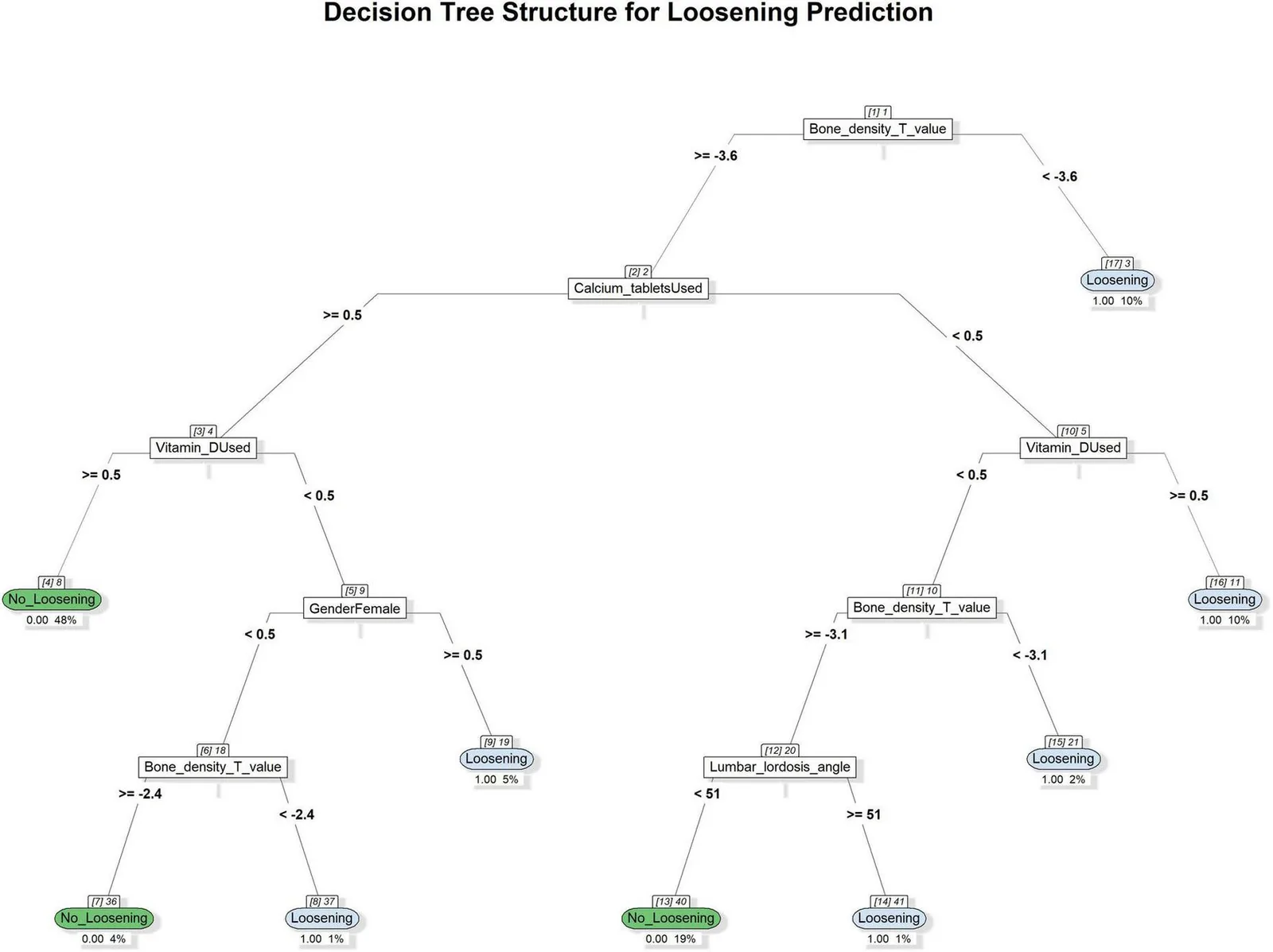
Structure of the decision tree model for screw loosening prediction. This decision tree was constructed with screw loosening as the outcome variable, incorporating key predictors including bone mineral density T-score, calcium tablet use, vitamin D use, gender, and lumbar lordosis angle. The root node was bone mineral density T-score (cutoff = −3.6); patients with a T-score < −3.6 were directly classified as high-risk for screw loosening, while those with T-score ≥ −3.6 were further stratified by calcium tablet use. Subsequent nodes sequentially split by vitamin D use, gender, and lumbar lordosis angle. Terminal nodes labeled the outcome (green for no loosening, blue for loosening) and the corresponding sample proportion, clearly illustrating the interactions of variables and risk stratification pathways.

The classification decision tree model constructed based on the optimal feature set is shown in Figure 7. The results demonstrated that bone mineral density T-score was the primary stratification variable, followed by calcium tablet use, vitamin D use, sex, and lumbar lordosis angle. Through stepwise threshold partitioning, the model clearly identified high-risk pathways for implant loosening:

*Pathway 1 (extremely high risk)*: BMD T-score < −3.6 → all patients in this node (*n* = 42, 100%) experienced loosening in this dataset.
*Pathway 2 (high risk)*: BMD T-score ≥ −3.6 and no calcium tablet use → further stratified by vitamin D use: patients not taking vitamin D had an extremely high risk of loosening, whereas those taking vitamin D had a moderate risk.
*Pathway 3 (moderate risk)*: BMD T-score ≥ −3.6 and calcium tablet use → stratified by sex: male patients tended to have no loosening; for female patients, the risk increased significantly when lumbar lordosis angle was ≥ 51°.

The decision tree intuitively illustrates that severely reduced bone mineral density, absence of calcium and vitamin D supplementation, female sex, and increased lumbar lordosis angle are key combined features associated with loosening risk.

### Individualized nomogram

**FIGURE 8:**
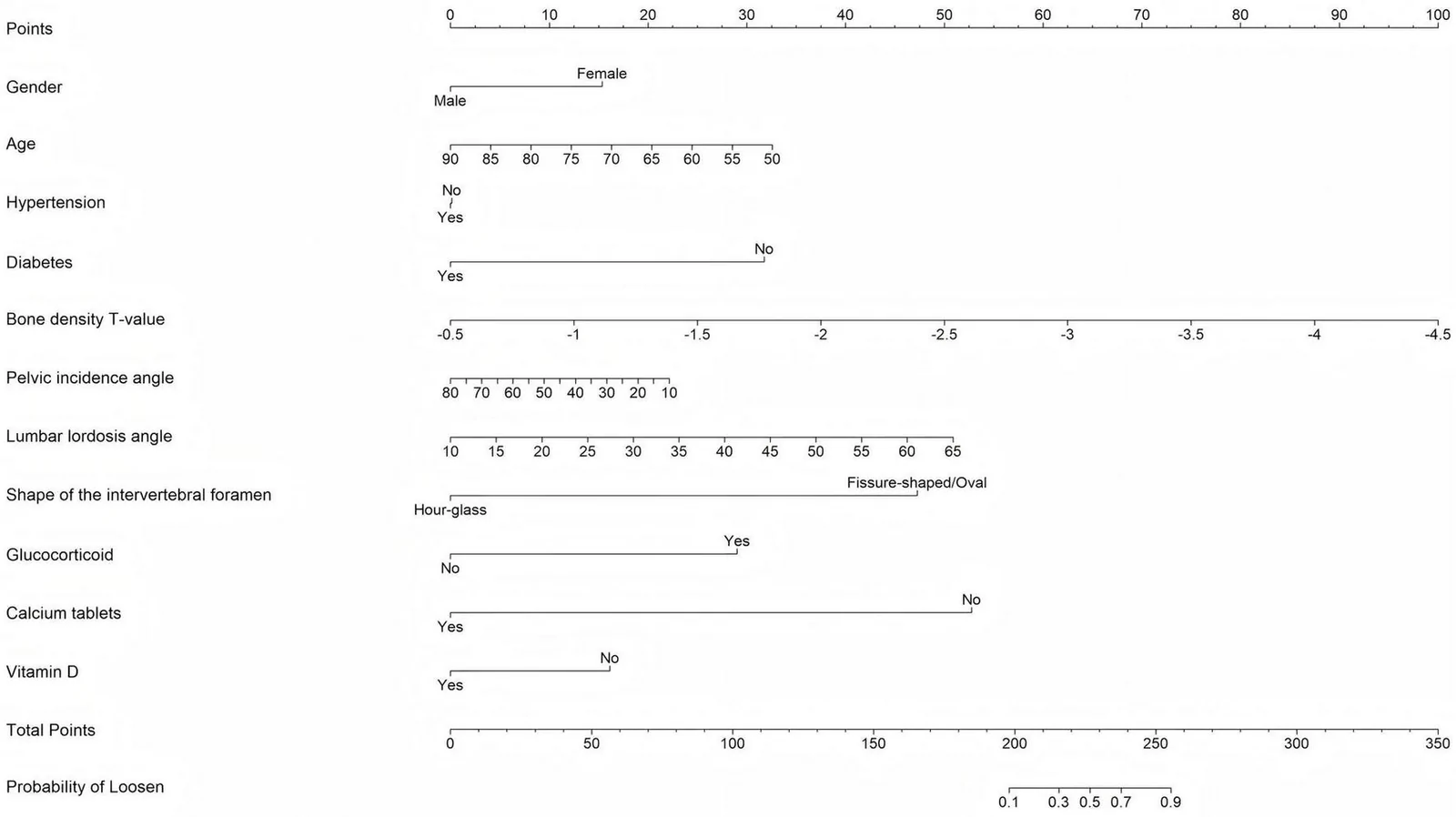
Nomogram for predicting the risk of screw loosening after lumbar surgery. This nomogram was developed based on independent predictors selected by LASSO regression, for individualized prediction of screw loosening risk. Each predictor (gender, age, hypertension, diabetes, bone mineral density T-score, pelvic incidence angle, lumbar lordosis angle, intervertebral foramen shape, glucocorticoid use, calcium tablet use, and vitamin D use) corresponds to a score axis. For each patient, the score of each variable is obtained by projecting the value upward to the “Points” scale. The total points are summed and projected downward to the “Probability of Loosen” scale at the bottom, to directly read the predicted probability of screw loosening. The nomogram visually quantifies the weight of each factor, with bone mineral density T-score, calcium tablet use, and intervertebral foramen shape contributing most significantly to the prediction probability.

The nomogram for predicting the risk of implant loosening, constructed based on the multivariate logistic regression model, is shown in Figure 8. The model included 11 predictors: sex, age, hypertension, diabetes, bone mineral density T-score, pelvic incidence angle, lumbar lordosis angle, intervertebral foramen morphology (with hour-glass shape as the reference), glucocorticoid use, calcium tablet use, and vitamin D use. The model showed good goodness-of-fit (Nagelkerke *R*^2^ = 0.708, Hosmer–Lemeshow test *P* > 0.05) and excellent discrimination (AUC = 0.939).

The scoring pattern of the nomogram is as follows: female sex, older age, presence of hypertension or diabetes, long-term glucocorticoid use, decreased bone mineral density T-score, increased lumbar lordosis angle, fissure-shaped or oval intervertebral foramen (relative to hour-glass shape), no calcium tablet use, and no vitamin D supplementation each contributed to a higher individual point score, indicating an increased risk of loosening; opposite conditions resulted in lower point scores. The contribution of each variable to the total score was consistent with the LASSO selection and decision tree analysis, with bone mineral density T-score, calcium tablet use, and intervertebral foramen morphology (hour-glass shape) having the greatest impact on the total score.

Clinical application steps: First, for each patient, locate the corresponding value on each variable axis, then draw a vertical line upward to the Points axis to obtain the individual score for that variable. Second, sum the scores of all 11 variables to obtain the Total Points. Finally, locate the cumulative value on the Total Points axis and draw a vertical line downward to the Probability axis to read the individual predicted probability of postoperative implant loosening.

Internal validation was performed using the bootstrap method (1,000 resamples). The calibration curve showed that the predicted probabilities were highly consistent with the observed probabilities (Hosmer–Lemeshow test *P* = 0.372). The nomogram is highly interpretable and convenient for clinical application, helping to identify high-risk patients preoperatively and to develop individualized intervention strategies.

## Discussion

In this multicenter study of 630 osteoporotic patients undergoing PLIF, we used LASSO logistic regression to select 11 independent predictors, constructed and compared four machine learning models, identified the decision tree as the best performing model (test set AUC = 0.971, sensitivity 0.889, specificity 0.941), and developed an individualized nomogram (concordance index = 0.928). The present study adds to the existing literature by integrating multidimensional variables—including clinical characteristics, imaging parameters [pelvic incidence (PI), lumbar lordosis (LL), foraminal morphology], and anti-osteoporosis medication history (calcium, vitamin D, glucocorticoids)—within a multicenter cohort, systematically comparing several machine learning approaches for predicting pedicle screw loosening, and achieving complementary use of a visualized decision tree and a nomogram.

In our study, bone mineral density T-score was the primary splitting variable in the decision tree, and a T-score < −3.6 was associated with a very high probability of loosening (>99%), which is consistent with previous reports. Du et al. found that a pedicle HU value < 106.32 was an independent risk factor for screw loosening (HU is strongly positively correlated with T-score); Götschi et al. (12) using finite element analysis, also confirmed that CT-derived bone density parameters are central determinants of screw mechanical stability. Our study exploratorily identified a specific clinical threshold (−3.6) that is substantially lower than the WHO diagnostic threshold for osteoporosis (−2.5), suggesting that patients with a severely low T-score (<-3.6) may be considered potential candidates for cement augmentation. However, this threshold was derived from a single data-driven analysis and should be viewed as hypothesis-generating rather than definitive. It requires prospective external validation before clinical implementation (31, 32).

Calcium supplementation (coefficient −1.526) was the factor most strongly associated with lower risk, and vitamin D supplementation (−0.42) was also associated with significantly lower risk. The decision tree revealed a combined protective effect of calcium and vitamin D: among patients with T-score ≥ −3.6, those who did not take calcium and also lacked vitamin D had a very high risk, whereas those who took both had the lowest risk. Previous predictive models (e.g., the PSL score) rarely included anti-osteoporosis medication history; our study addresses this gap. Hour-glass foraminal morphology (coefficient −0.92) was the second most important protective factor—a finding that has not been previously reported. This may reflect preserved local anatomic integrity of the pedicle–vertebral body complex or may limit excessive intraoperative decompression, thereby protecting screw purchase (31, 33).

Glucocorticoid use (0.573) was the strongest risk factor, consistent with the classical mechanism of glucocorticoid–induced osteoporosis. Among spinopelvic parameters, LL (coefficient 0.28) was identified as a risk factor, which aligns with the broader literature linking spinopelvic parameters (e.g., high PI) to screw loosening (14). Female sex (0.31) was a risk factor, consistent with the epidemiology of osteoporosis. Hypertension and diabetes appeared as protective factors (coefficients −0.48 and −0.65, respectively), which is contrary to expectation. One speculative explanation is that patients with these comorbidities might have received more structured medical follow-up, potentially including more regular anti-osteoporosis treatment, or might have lower physical activity levels, reducing biomechanical stress on the screws (34, 35). However, this finding may also be due to residual confounding or chance. Given the lack of supporting evidence, it should be interpreted with caution and warrants investigation in future prospective studies.

Regarding model performance, our decision tree (test set AUC = 0.971) outperformed several published models: the HU-based model by Du et al. (AUC = 0.766), the PSL risk score (AUC = 0.887), and the finite element-based model by Götschi et al. (AUC = 0.919). The improved performance likely reflects the inclusion of a more comprehensive set of predictors (especially anti-osteoporosis medication history and spinopelvic parameters), the use of LASSO to avoid multicollinearity, and the ability of the decision tree to automatically capture nonlinear interactions (e.g., the combined effect of calcium and vitamin D). Compared with other machine learning models, naïve Bayes assumes conditional independence of features—an assumption that was violated because variables such as bone density T-score and calcium use, or PI and LL, were correlated, leading to its lower AUC (0.871). LDA assumes multivariate normality and equal covariance matrices, which may not have been fully satisfied by our data. Logistic regression was robust (AUC = 0.926) but, being a linear function, cannot inherently reveal hierarchical relationships. Thus, the decision tree achieved both high accuracy and high interpretability in our setting: its tree structure clearly identified the “absolute indication” group (T*score < −3.6), revealed the hierarchical role of modifiable factors (combined protection of calcium and vitamin D), and identified a refined combination of features (women taking calcium but with LL ≥ 51° have significantly elevated risk). Although the finite element–based model by Götschi et al. achieved a high AUC, it requires specialized software, limiting routine clinical adoption. The PSL risk score is simpler but has a lower AUC and did not include anti-osteoporosis medication history. Our decision tree offers a favorable balance between performance and ease of use (36).

The nomogram, as a visualization of logistic regression, provides a continuous probability output and is suitable for quantitative risk assessment during preoperative counseling. Its C-index was 0.928 and calibration was good (Hosmer–Lemeshow test *P* = 0.372). The decision tree, by contrast, provides discrete classification rules and is suitable for rapid screening in outpatient settings. Both tools are based on the same set of variables, yield highly consistent results, and are complementary. Such a dual–tool strategy has rarely been reported in spine surgery prediction models and represents a novel methodological approach (37).

Our study has several limitations. First, despite the multicenter design, the retrospective nature may introduce selection bias and information bias. Second, although the dataset was derived from three centers, the validation was based on an internal split-sample approach rather than a fully independent external cohort. External temporal or geographic validation has not yet been performed, and further expansion to more centers is needed. Third, the data on calcium and vitamin D use relied on medical history recall, which may be subject to recall bias; the dosage, frequency, and adherence were not recorded—we only used a binary “yes/no” classification, which may have resulted in some loss of information. Fourth, decision trees are known to be sensitive to small data changes (instability); different training-test splits may generate slightly different tree structures. Although we partially mitigated this through stratified random sampling and cross-validation, and further confirmed model stability via the complexity and learning curves (Supplementary Figures 1A,B), external data are needed to confirm stability. Fifth, the outcome of this study was the occurrence of pedicle screw loosening, which was assessed based on imaging findings; however, the prediction model was developed using a relatively limited sample size. Larger multicenter datasets are required to further refine and externally validate the model. Future studies should conduct multicenter prospective cohorts, develop web-based interactive calculators, explore deep learning models to automatically extract radiomic features, and design pragmatic clinical trials to evaluate whether model-guided preoperative optimization (e.g., intensified anti-osteoporosis therapy, calcium and vitamin D supplementation) or intraoperative strategies (e.g., cement-augmented screws, alternative fixation techniques) can reduce the incidence of postoperative screw loosening (13, 38).

Additionally, the decision tree model was developed with 180 outcome events and 11 candidate predictors, yielding an events–per–variable (EPV) ratio of approximately 16, which meets the minimum recommended threshold of 10. Nonetheless, the tree structure (depth of 5) carries a risk of overfitting, and the identified splitting thresholds (e.g., T-score < –3.6) are data–derived and require external validation before clinical implementation.

Despite these limitations, this study has several important clinical implications. It identifies 11 easily obtainable preoperative predictors—among which calcium/vitamin D use, foraminal morphology, and spinopelvic parameters have been rarely included in previous models—thereby broadening the scope of preoperative risk assessment for pedicle screw loosening. The complementary use of a decision tree and a nomogram addresses both rapid screening and detailed quantitative evaluation. Based on our multicenter results, we suggest that all osteoporotic patients scheduled for PLIF undergo preoperative assessment of bone mineral density T-score. A T-score < −3.6 should raise suspicion of subsequent screw loosening, prompting preoperative optimization (e.g., intensified anti-osteoporosis therapy) and consideration of intraoperative strategies such as cement-augmented screws or alternative fixation techniques. However, this threshold was derived from our dataset and requires external validation before it can be used as a definitive clinical cutoff (22, 33).

## Conclusion

In this multicenter study of 630 osteoporotic patients, LASSO logistic regression identified 11 independent predictors of pedicle screw loosening after PLIF. Among four machine learning models, the decision tree performed best (test set AUC = 0.971, sensitivity 0.889, specificity 0.941). Its visualized tree structure revealed that a bone mineral density T-score < −3.6, lack of calcium and vitamin D supplementation, female sex, and lumbar lordosis (LL) ≥ 51° are key combined risk factors for postoperative screw loosening. The multivariable logistic regression-based nomogram (C-index = 0.928) provides individualized probability estimation of loosening risk. The two tools are complementary and consistent with the LASSO results. Nevertheless, these findings are derived from an internal split-sample validation and should be considered preliminary until confirmed by independent external cohorts. The decision tree and nomogram can rapidly identify high-risk patients preoperatively, thereby informing early preoperative optimization (e.g., intensified anti-osteoporosis therapy, calcium and vitamin D supplementation) and intraoperative strategies such as cement-augmented screws or alternative fixation techniques. The multicenter design enhances generalizability, but prospective external validation is still warranted.

## Data Availability

The raw data supporting the conclusions of this article will be made available by the authors, without undue reservation.

## References

[1] Salari N Darvishi N Bartina Y Larti M Kiaei A Hemmati M et al. Global prevalence of osteoporosis among the world older adults: a comprehensive systematic review and meta-analysis. *J Orthop Surg.* (2021) 16:669. 10.1186/s13018-021-02821-8 34774085 PMC8590304

[2] Xiao P-L Cui A-Y Hsu C-J Peng R Jiang N Xu X-H et al. Global, regional prevalence, and risk factors of osteoporosis according to the World Health Organization diagnostic criteria: a systematic review and meta-analysis. *Osteoporos Int J Establ Result Coop Eur Found Osteoporos Natl Osteoporos Found USA.* (2022) 33:2137–53. 10.1007/s00198-022-06454-3 35687123

[3] GBD 2021 Low Bone Mineral Density Collaborators. The global, regional, and national burden attributable to low bone mineral density, 1990-2020: an analysis of a modifiable risk factor from the Global Burden of Disease Study 2021. *Lancet Rheumatol.* (2025) 7:e873–94. 10.1016/S2665-9913(25)00105-5 40972625 PMC12623303

[4] Wang L Yu W Yin X Cui L Tang S Jiang N et al. Prevalence of osteoporosis and fracture in China: the China osteoporosis prevalence study. *JAMA Netw Open.* (2021) 4:e2121106. 10.1001/jamanetworkopen.2021.21106 34398202 PMC8369359

[5] Lyu FF Ramoo V Chui PL Ng CG Zhang Y. Prevalence rate of primary osteoporosis in China: a meta-analysis. *BMC Public Health.* (2024) 24:1518. 10.1186/s12889-024-18932-w 38844897 PMC11155107

[6] Odén A McCloskey EV Kanis JA Harvey NC Johansson H. Burden of high fracture probability worldwide: secular increases 2010-2040. *Osteoporos Int J Establ Result Coop Eur Found Osteoporos Natl Osteoporos Found USA.* (2015) 26:2243–8. 10.1007/s00198-015-3154-6 26018089

[7] Oumer KS Liu Y Yu Q Wu F Yang S. Awareness of osteoporosis among 368 residents in China: a cross-sectional study. *BMC Musculoskelet Disord.* (2020) 21:197. 10.1186/s12891-020-03217-1 32228605 PMC7106623

[8] Wu Y Lai Z Yan H Zhou B Chen W Zeng Z. [Trends in burden of osteoporosis in China from 1990 to 2023 and analysis of epidemiological factors]. *Zhongguo Xiu Fu Chong Jian Wai Ke Za Zhi Zhongguo Xiufu Chongjian Waike Zazhi Chin J Reparative Reconstr Surg.* (2026) 40:378–86. 10.7507/1002-1892.202601066 41839551 PMC12991889

[9] Odland K Pottinger TJ Grund PM Polly DW. S1 pedicle screw loosening: a systematic review and meta-analysis of risk factors and outcomes. *Int J Spine Surg.* (2025) 19:426–36. 10.14444/8773 40883010 PMC12570065

[10] Wang H Xiao C Zhang K Xie M Li S. Clinical risk factors associated with cage migration after posterior approaches for lumbar interbody fusion: a 10-year systematic review and meta-analysis. *Eur Spine J Off Publ Eur Spine Soc Eur Spinal Deform Soc Eur Sect Cerv Spine Res Soc.* (2025) 34:4178–87. 10.1007/s00586-025-09109-z 40619527

[11] Du P Liang M Chen R Wang T Fan N Yuan S et al. Comparison of the predictive roles of CT- and MRI-based pedicle regional osteoporosis status measurements for pedicle screw loosening after posterior lumbar interbody fusion. *Glob Spine J.* (2026) 16:1075–84. 10.1177/21925682251371602 40879064 PMC12397096

[12] Götschi T Maranta G Fasser M-R Farshad M Widmer J. Validation of an automated spinal fusion finite element modeling framework for the prediction of pedicle screw loosening. *J Biomech.* (2025) 192:112967. 10.1016/j.jbiomech.2025.112967 40974780

[13] Huang Y-C Liu P-C Lin H-H Wang S-T Su Y-P Chou P-H et al. Risk prediction model of pedicle screw loosening within 2 years after decompression and instrumented fusion surgery for degenerative lumbar disease. *Spine J Off J North Am Spine Soc.* (2025) 25:1474–82. 10.1016/j.spinee.2025.01.033 39894275

[14] Siribumrungwong K Satthanan S Wuttiworawanit B Pinitchanon P Suntharapa T. Effect of high pelvic incidence on fixation failure in single-level transforaminal lumbar interbody fusion for low-grade spondylolisthesis: a retrospective cohort study. *J Clin Med.* (2026) 15:3199. 10.3390/jcm15093199 42122932 PMC13164481

[15] Hoffman H Sims JJ Inoa-Acosta V Hoit D Arthur AS Draytsel DY et al. Machine learning for clinical outcome prediction in cerebrovascular and endovascular neurosurgery: systematic review and meta-analysis. *J Neurointerventional Surg.* (2025) 17:661–7. 10.1136/jnis-2024-021759 38772570

[16] Wang P Liu L Xie Z Ren G Hu Y Shen M et al. Explainable machine learning models for prediction of surgical site infection after posterior lumbar fusion surgery based on shapley additive explanations. *World Neurosurg.* (2025) 197:123942. 10.1016/j.wneu.2025.123942 40154601

[17] Fasser M-R Gerber G Passaplan C Cornaz F Snedeker JG Farshad M et al. Computational model predicts risk of spinal screw loosening in patients. *Eur Spine J Off Publ Eur Spine Soc Eur Spinal Deform Soc Eur Sect Cerv Spine Res Soc.* (2022) 31:2639–49. 10.1007/s00586-022-07187-x 35461383

[18] Joshi RS Lau D Ames CP. Machine learning in spine surgery: predictive analytics, imaging applications and next steps. *Semin Spine Surg.* (2021) 33:100878. 10.1016/j.semss.2021.100878

[19] WHO. *Assessment of Fracture Risk and Its Application to Screening for Postmenopausal Osteoporosis. Report of a WHO Study Group. (WHO Technical Report Series; No. 843).* Geneva: World Health Organization (1994). p. 1–129. 7941614

[20] Kanis JA. Diagnosis of osteoporosis and assessment of fracture risk. *Lancet.* (2002) 359:1929–36. 10.1016/S0140-6736(02)08761-5 12057569

[21] Aghayev E Zullig N Diel P Dietrich D Benneker LM. Development and validation of a quantitative method to assess pedicle screw loosening in posterior spine instrumentation on plain radiographs. *Eur Spine J Off Publ Eur Spine Soc Eur Spinal Deform Soc Eur Sect Cerv Spine Res Soc.* (2014) 23:689–94. 10.1007/s00586-013-3080-2 24170238 PMC3940783

[22] Galbusera F Volkheimer D Reitmaier S Berger-Roscher N Kienle A Wilke H-J. Pedicle screw loosening: a clinically relevant complication? *Eur Spine J Off Publ Eur Spine Soc Eur Spinal Deform Soc Eur Sect Cerv Spine Res Soc.* (2015) 24:1005–16. 10.1007/s00586-015-3768-6 25616349

[23] Spirig JM Sutter R Götschi T Farshad-Amacker NA Farshad M. Value of standard radiographs, computed tomography, and magnetic resonance imaging of the lumbar spine in detection of intraoperatively confirmed pedicle screw loosening-a prospective clinical trial. *Spine J Off J North Am Spine Soc.* (2019) 19:461–8. 10.1016/j.spinee.2018.06.345 29959101

[24] Ali Baig R Quiceno E Soliman MAR Aguirre AO Okai BK Kuo CC et al. Definition of cage subsidence in transforaminal lumbar interbody fusion (TLIF) approach and posterior lumbar interbody fusion (PLIF) approach - a systematic review. *J Clin Neurosci Off J Neurosurg Soc Australas.* (2025) 133:111048. 10.1016/j.jocn.2025.111048 39827769

[25] Legaye J Duval-Beaupère G Hecquet J Marty C. Pelvic incidence: a fundamental pelvic parameter for three-dimensional regulation of spinal sagittal curves. *Eur Spine J Off Publ Eur Spine Soc Eur Spinal Deform Soc Eur Sect Cerv Spine Res Soc.* (1998) 7:99–103. 10.1007/s005860050038 9629932 PMC3611230

[26] Duval-Beaupère G Schmidt C Cosson P. A Barycentremetric study of the sagittal shape of spine and pelvis: the conditions required for an economic standing position. *Ann Biomed Eng.* (1992) 20:451–62. 10.1007/BF02368136 1510296

[27] Lee CK Rauschning W Glenn W. Lateral lumbar spinal canal stenosis: classification, pathologic anatomy and surgical decompression. *Spine.* (1988) 13:313–20. 10.1097/00007632-198803000-00015 3388117

[28] Lee S Lee JW Yeom JS Kim K-J Kim H-J Chung SK et al. A practical MRI grading system for lumbar foraminal stenosis. *AJR Am J Roentgenol.* (2010) 194:1095–8. 10.2214/AJR.09.2772 20308517

[29] Yan S Wang K Zhang Y Guo S Zhang Y Tan J. Changes in L4/5 intervertebral foramen bony morphology with age. *Sci Rep.* (2018) 8:7722. 10.1038/s41598-018-26077-1 29769656 PMC5955929

[30] Özer AF Akyoldaş G Çevik OM Aydın AL Hekimoğlu M Sasani M et al. Lumbar foraminal stenosis classification that guides surgical treatment. *Int J Spine Surg.* (2022) 16:666–73. 10.14444/8311 35710724 PMC9421203

[31] Cho JH Hwang CJ Kim H Joo Y-S Lee D-H Lee CS. Effect of osteoporosis on the clinical and radiological outcomes following one-level posterior lumbar interbody fusion. *J Orthop Sci Off J Jpn Orthop Assoc.* (2018) 23:870–7. 10.1016/j.jos.2018.06.009 30431006

[32] Zhao H Wang Y-J Wang R-G Liu D Duan Y-Q Liu Y-J et al. Three-dimensional hounsfield units measurement of pedicle screw trajectory for predicating screw loosening in lumbar fusion surgery. *Clin Interv Aging.* (2023) 18:485–93. 10.2147/CIA.S389059 37008803 PMC10065021

[33] Mo G-Y Guo H-Z Guo D-Q Tang Y-C Li Y-X Yuan K et al. Augmented pedicle trajectory applied on the osteoporotic spine with lumbar degenerative disease: mid-term outcome. *J Orthop Surg.* (2019) 14:170. 10.1186/s13018-019-1213-y 31171020 PMC6555715

[34] Zhang C Wen T Li C Ruan D He Q. Cluster phenomenon of vertebral refractures after posterior pedicle screw fixation in a patient with glucocorticosteroid-induced Kümmell’s disease: a treatment dilemma. *Arch Osteoporos.* (2021) 16:93. 10.1007/s11657-021-00941-6 34105042

[35] Zhou Q Zhang J-X Zheng Y-F Teng Y Yang H-L Liu H et al. Effects of different pedicle screw insertion depths on sagittal balance of lumbar degenerative spondylolisthesis, a retrospective comparative study. *BMC Musculoskelet Disord.* (2021) 22:850. 10.1186/s12891-021-04736-1 34615516 PMC8493756

[36] Jiang F Li X Liu L Xie Z Wu X Wang Y. Automated machine learning-based model for the prediction of pedicle screw loosening after degenerative lumbar fusion surgery. *Biosci Trends.* (2024) 18:83–93. 10.5582/bst.2023.01327 38417874

[37] Zhang Y Li Y Hai Y Guan L Zhang X Pan A et al. A nomogram for predicting screw loosening after single-level posterior lumbar interbody fusion utilizing cortical bone trajectory screw: a minimum 2-year follow-up study. *Front Surg.* (2022) 9:950129. 10.3389/fsurg.2022.950129 36311946 PMC9615560

[38] Sushmitha, Nayak SG Kanthi M Bhat NS. Significance of prediction techniques in spinal fusion surgery. *2022 International Conference on Disruptive Technologies for Multi-Disciplinary Research and Applications (CENTCON).* (2022). p. 206–10. 10.1109/CENTCON56610.2022.10051196

